# Progress in Surgery of the Brain and Nerves

**Published:** 1902-10-04

**Authors:** 


### Progress in Surgery of the Brain and Nerves.

(*Continued from page* 453.)

**Cerebral Abscess.**—Herbert F. Waterhouse[8] records a successful case occurring in a woman æt. 26, who had had right otorrhœa since infancy. She had had severe headache for nine days. When he saw her she was comatose, pulse 44, temperature 100°; slight vomiting had been present, right pupil larger than left, commencing optic neuritis. There were no localising symptoms. Owing to her condition her gait could not be examined. She was trephined over the cerebellum on the right side, but no pus found, then over the temporo sphenoidal lobe, when pus was evacuated. The writer draws attention to great difficulty in deciding whether an intracranial abscess is in the cerebrum or cerebellum. He points out the danger of waiting in such cases, as if left too long operation will probably be too late, and in unrelieved cases of intracranial abscess sudden death is not an infrequent termination. Another successful case of cerebral abscess is recorded by Albert Lucas.[9] The patient, a woman æt. 45, was the subject of double otorrhœa for 18 years. She had had severe headache for a week, referred to the right side of the head, nausea but no vomiting, no optic neuritis, paralysis of the left side. She had nocturnal delirium. Pulse 68, temp. subnormal. After a long search, pus was found about 1½ inch from the brain surface, and apparently in the region of the ascending parietal convolution. Except for deafness and otorrhœa, which were present before the operation, the woman is now quite well. J. S. M'Ardle[10] relates six successful cases of trephining for intracranial hæmorrhage of traumatic origin. In two of them there was an interval of consciousness and relapse into coma; the other four did not relapse into unconsciousness, two of them developed fits, the other two paralysis. In this connection an interesting case is recorded by Dr. Pollock.[11] A man fell from his horse in Australia, and was unconscious for two hours; he then recovered and mounted his horse, riding home a distance of nine miles. He then relapsed into coma, and surgical assistance being unattainable, he remained unconscious for three weeks. He recovered consciousness, and for some time had epileptic fits daily. He then travelled to London, but after starting on the journey the fits ceased, and he has remained well since. An interesting case of severe cranial injury, with recovery, is recorded by Vaughan.[12] A girl æt 20, sustained a compound depressed fracture of the entire frontal bone and fracture of the nasal bones; after the bones had been replaced she recovered, but has completely lost the sense of smell. She has since been subject to periodic attacks of headache and insomnia, associated with depression and excitability. The same writer narrates the case of a youth who received a blow from a club on the head; there was no fracture of the skull, and not much external injury. The post-mortem revealed no fracture of the skull, but extensive laceration of the brain and hæmorrhage. He cites this case as an example of the great elasticity of the skull. Two cases are recorded bearing on the connection of the occipital lobes with the visual fields; in one, under the care of Mr. Willet,[13] the patient sustained a compound depressed fracture of the left occipital region with laceration of the left occipital lobe. He complained afterwards of inability to see objects to his right. The perimeter showed a partial right hemianopia, the lower quadrants of the right visual fields being lost. Ridley[14] records the other case: the occipital lobes were injured by shrapnel and there ensued considerable contraction of the visual fields. The former of these two cases recovered almost completely, the latter apparently has not shown much improvement. James Laurie[15] has removed a bullet from the occipital region which he previously localised by means of a skiagram. The missile entered through the left frontal region. The patient, a boy of 15, had right-sided paralysis and aphasia, passing later into coma. A No. 8 Jacque's catheter was used as a drain, passed all along the track, and brought out through an opening in the occipital bone. The trephine discs were replaced and survived. The patient recovered from all his symptoms. Rushton Parker[16] records a case of epilepsy following upon an old compound depressed fracture of the skull in the left fronto-parietal region. The accident occurred in 1884; the patient was trephined at the time, he was under treatment for ten months with paralysis of the right arm, leg, and side of the face—this passed off. Three years later he had some fits which after a time ceased. In 1894 he had a return of fits, and a cystic translucent condition of the scar then existed. Mr. Parker excised the scar and brought the edge s of the scalp together; this gave him relief until 1901, when the fits returned together with mental disturbances The scar was again operated on, and was found to be adherent to the brain. The dura was deficient over the old trephine hole. A piece of fairly stiff gold foil was interposed between the dura and skull, covering over the hole, and the skin sutured up. The patient made a good recovery and has remained well since.

**Spina Bifida.**—In treatment the views of surgeons have turned very strongly of late to the advisability of excision to the exclusion of the older methods, notably the injection of irritants. In either method there is considerable risk attending the operation. In excision, fatal shock and collapse at the time, presumably due to the sudden escape of cerebro-spinal fluid, also, in some cases, apparently due to the manipulation of nerves and cord, and leakage afterwards, giving rise to sepsis. It is advisable to defer the operation as long as possible, as the older the child the better is it able to stand the operation. In the treatment by injection there is usually severe constitutional reaction afterwards and sometimes fatal collapse at the time, and the method is by no means a certain cure. We have not yet any very reliable statistics by which to compare methods. Several cases have lately been recorded. James H. Nicoll[17] has operated on a series of ten cases in the out-patient department of the Glasgow Children's Hospital, with one death, and that from carboluria. The children were allowed to go home immediately afterwards and were visited occasionally by a surgical nurse. The ages varied from two weeks to five months. He does not refer to the question of postponing the operation until the child is older, but some of the cases, he says, were thin-walled and could not be left. He lays open the sac freely and does not mention any precautions taken to prevent the too sudden or free escape of fluid, but he has not recorded any untoward results from this cause—his series of cases have been very successful. J. W. Henson[18] contributes an article on this subject. He lays great stress on the danger attending the escape of cerebro-spinal fluid and gives detailed instruction for the prevention of its occurrence. He records one case of fatal collapse after opening the sac. He finds that handling the nerves or cord, when it is necessary to dissect them from the sac, causes considerable shock, and he has consequently abandoned this practice and returns them together with the sac, which he splits up by longitudinal cuts between the nerve cords. He strongly condemns the treatment by injection, except as a last resort in quite inoperable cases. G. L. St. George[19] records two cases somewhat similar to each other. One a child æt. ten days, was treated by Morton's fluid which gave rise to considerable shock and fever. The injection was repeated after a few days with less disturbance and finally the sac partially shrivelled up, except in one part which he finally excised. In the other case, a child æt. seven weeks, he excised the sac; the patient recovered without an untoward symptom. Dr. John Lithgow[20] has successfully removed a meningocele from a child ten months old, also a large encephalocele as big as the patient's head from another child twelve days old; the latter child did well for a fortnight and then died of convulsions. Another writer[21] records a case of an occipital meningocele considerably larger than the child's head which he removed from an infant four months old, the patient did not recover.

8 Clin. Jour., November. 9 Birmingham Med. Rev., November 10 Dub. Jour. Med. Sc., December. 11 Ibid. 12 Amer. Jour. Med. Sci., November. 13 Lancet, October 12. 14 Practitioner, April. 15 Scot. Med. Soc. Jour., February. 16 Brit. Med. Jour., May 24. 17 Ibid., June 21. 18 Charlotte Med. Jour., December. 19 Lancet, March 15. 20 Brit. Med. Jour., January. 21 Lancet, January 11.

